# Editorial: Innovations in Research and Practice of Family Based Treatment for Eating Disorders

**DOI:** 10.3389/fpsyt.2020.638503

**Published:** 2021-01-21

**Authors:** James Lock, Daniel Le Grange, Dasha Nicholls, Jennifer Couturier

**Affiliations:** ^1^Department of Psychiatry, Stanford University, Stanford, CA, United States; ^2^Department of Psychiatry, University of California, San Francisco, San Francisco, CA, United States; ^3^Department of Psychiatry, Imperial College London, London, United Kingdom; ^4^Department of Psychiatry, McMaster University, Hamilton, ON, Canada

**Keywords:** Family-based Treatment, research, clinical, eating disorders, anorexia nervosa, bulimia nervosa

This special issue of *Frontiers in Psychiatry/Psychosomatic Medicine* is focused on innovations in research and treatment for eating disorders using Family-based Treatment (FBT). FBT is at this time the first line treatment recommended by most international treatment guidelines for Anorexia Nervosa (AN) and Bulimia Nervosa (BN) for adolescents (1). Nonetheless, there is still much to be learned about FBT and this special issue provides a broad view of the clinical and research directions that are taking place currently. There are 15 articles in this special issue covering a range of topics from neuroscience of FBT, research protocols of current ongoing studies, and novel clinical applications and adaptations. Contributions are from scientists and clinicians from around the world including Australia, New Zealand, the UK, Sweden, Canada and the US.

Starting first with novel clinical applications, Spettigue, Norris et al. in their article entitled “Feasibility of Implementing a Family-Based Inpatient Program for Adolescents With Anorexia Nervosa: A Retrospective Cohort Study” describe how the principles of FBT can be adapted to an inpatient setting. This is an important contribution because it highlights that although FBT is an outpatient treatment, many of the key aspects of the approach—most importantly parental involvement—can be helpful in more intensive programmatic settings. Loeb et al. report on a pilot study of adapted FBT for youth at risk AN. This study suggests that FBT as well as other interventions are potentially useful in treating eating disorder in children and adolescent before they become highly symptomatic. Early intervention for at risk youth is an important avenue of research and aims at secondary prevention. As such, this study adds to the body of prevention and related early intervention clinical and research endeavors. In a similar vein, Spettigue, Aldaqqaq et al. report outcomes of a small case series of children and adolescents with mild eating disorder symptoms in their article “A Brief Modified Family-based Treatment Intervention for Youth with Mild Eating Disorders: A Case Series.” These authors found that a very brief FBT-like intervention led to symptomatic improvement in these sub-clinical cases. The brevity of the intervention adds to its potential as a secondary prevention approach as it likely increases feasibility and acceptability.

Several articles focus on broader overviews of how FBT might be helpful. Lock and Nicholls describe how implementing FBT necessarily involves recognizing that the initial narrow behavioral focus of FBT on weight restoration broadens over the course of treatment to include adolescent developmental issues as treatment progresses. This allows FBT to address broader clinical issues that often accompany adolescents with AN, including problems that might be understood as comorbidities. Adding a neuropsychiatric understanding about the underpinnings of exposure and response prevention—one way to conceptualize parental re-feeding efforts—is described as a critical aspect of FBT related to brain neuroscience in the contribution by Mysliwiec entitled “Neuroscience of Adolescent Anorexia Nervosa: Implications for Family-based Treatment.” This theoretical description of how FBT interventions “change the brain” makes for provocative and inspiring reading. In addition, Lavender in her article “Rebooting ‘failed' Family-based Treatment” describes how cases that appear not to have responded to standard FBT can be successfully treated by greater adherence and fidelity to the treatment approach and manual. This article serves as a kind of corrective to the notion that once FBT fails, it is not possible to re-visit the approach with motivated families.

Turning to contributions that describe new research protocols using FBT, Bohon et al. describe a study using fMRI to evaluate anatomical brain changes secondary to re-feeding as a possible mediator of outcome. These authors claim that understanding neural change during treatment with FBT, particularly in the early weeks, has the potential to improve outcome by enhancing motivation for rapid behavior change, while also examining possible mechanisms by which early treatment response leads to improved outcome. Another protocol currently being used in research is described by Wallin and Saha in their article “Implementation of Key Components of Evidence-based Family Therapy for Eating Disorders in Child and Adolescent Psychiatric Outpatient Care.” Their research evaluates whether implementing key components of family therapy during the first month of treatment in child and adolescent psychiatric outpatient care will lead to decreased inpatient utilization. Readers will likely find the design of these studies of interest, as well as anticipate future research findings from them.

There are several contributions related to broad themes of dissemination and implementation of FBT. For example, Le Grange et al. compared weight outcomes for adolescents with AN who received FBT in a randomized clinical research trial or non-research specialty care. They found no differences in outcome between the groups, suggesting that implementation of FBT in non-research settings can achieve results similar to those found in clinical trials. This finding should give confidence to clinical programs wishing to improve outcomes for patients in non-research settings. Hughes et al. in their contribution entitled “Adolescent and Parent Experience of Care at a Family-based Treatment Service for Eating Disorders” describes how patients and families treated with FBT on their clinical service experienced care. Their results found that parent and patients reported overall positive experiences of FBT. These findings are important because they counter the contention by some therapists and clinical programs concerned about the burden on families who receive FBT and resistance by adolescents, who they worry will not be cooperative with the approach. To examine the impact of therapists' attitudes on the uptake of FBT, Accurso et al. in their contribution “Attitudes toward Family-based Treatment Impact Therapists' Intent to Change Their Therapeutic Practice for Adolescent with Eating Disorders” found that providers reported very positive attitudes toward evidence-based practices in general and moderately positive attitudes toward FBT. Because attitudes of providers about treatment correlate with adoption and fidelity, these findings may indicate that FBT can be disseminated best by therapists who are supportive of the model.

There were, in addition, contributions that add to our understanding of what to expect in treatment using FBT. For example, Matheson et al., in their study entitled “Investigating Early Response to Treatment in a Multi-site Study of Adolescent Bulimia Nervosa” found markers of early response—reduction in purging at session 2, and binge eating at session 4—were related to abstinence of symptoms at the end of treatment, regardless of treatment type. Early response is potentially important because those who fail to achieve these markers might benefit from an adapted form of FBT or other treatments. Rosania and Lock in their article “Family-based Treatment for a Preadolescent with Avoidant/Restrictive Food Intake Disorder (ARFID)” describe a novel application of FBT to a younger population with ARFID. FBT for ARFID relies upon the same key interventions as FBT for AN, but the author identifies critical differences in the application of these interventions given the unique challenges of ARFID, particularly when characterized by sensory sensitivity. At this stage, FBT for ARFID needs further investigation and this case report is helpful in describing what might be needed for the approach to be effective for ARFID. Finally, Kimber et al. describe how clinicians can manage the unusual, but serious problem of child maltreatment in the context of FBT treatment in their article “Recognizing and Responding to Child Maltreatment: Strategies to Apply When Using Family-based Treatment for Eating Disorders.”

Taken together the collection of articles in this special issue offers clinicians and scientists an important opportunity to learn more about where things are with FBT and where they are going.

## Author Contributions

JL, DL, DN, and JC contributed to the composition of this editorial. All authors contributed to the article and approved the submitted version.

## Conflict of Interest

The authors declare that the research was conducted in the absence of any commercial or financial relationships that could be construed as a potential conflict of interest.
